# Transcriptome-wide association study and eQTL analysis to assess the genetic basis of bulb-yield traits in garlic (*Allium sativum*)

**DOI:** 10.1186/s12864-019-6025-2

**Published:** 2019-08-17

**Authors:** Siyuan Zhu, Xiaojun Chen, Xia Liu, Jian Zhao, Touming Liu

**Affiliations:** 1 Institute of Bast Fiber Crops and Center of Southern Economic Crops, Chinese Academy of Agricultural Sciences, Changsha, China; 2 Novogene Bioinformatics Institute, Beijing, China

**Keywords:** Garlic, Bulb yield, Association mapping, Expression quantitative trait locus (eQTL)

## Abstract

**Background:**

Garlic bulbs are abnormal expanding axillary buds that are rarely found among vascular plants. Bulb-yield is one of the valuable agronomic traits of garlic. However, due to the large genome size and a strictly asexual life cycle in the cultivars, the genetic basis of the yield traits are poorly understood in garlic.

**Results:**

In the present study, we carried out an association mapping for three yield traits of garlic bulbs: bulb weight (BW), diameter (BD), and the number of garlic cloves (CN), using the recently proposed transcriptome-referenced association study. In total 25, 2, and 30 single nucleotide polymorphisms (SNPs), were identified in the transcripts to be associated with BW, BD, and CN traits, respectively. Of the transcripts with associated SNPs, the expression of 17 of them showed a significant correlation with the corresponding traits in the population, suggesting their relation to bulbs yield traits. Six transcripts were long non-coding RNAs (lncRNAs), and the others encode proteins involved mainly in carbohydrate metabolism, transcription regulation, cytokinin activity, protein degradation, etc. In addition, expression quantitative trait locus (eQTL) and expression correlation analysis have revealed that seven CN-related transcripts displayed interrelation, constituting two potential pathways.

**Conclusion:**

This study provides novel insights into the genetic basis of the yield traits in garlic bulbs, and the identification of trait-associated SNPs/transcripts provides a basis for improving the bulb yield in garlic breeding.

**Electronic supplementary material:**

The online version of this article (10.1186/s12864-019-6025-2) contains supplementary material, which is available to authorized users.

## Background

Garlic (*Allium sativum* L.), one of the most economically important crops, is not only widely consumed as a condiment and green vegetable, but also has been associated with remarkable medicinal and nutraceutical properties [1, 2]. Owing to its notable values, garlic has been cultivated for more than 5000 years all over the world.

Despite the remarkable economic importance of garlic, the genetic basis of its agronomic traits is poorly understood. This lacunae in the knowledge can be associated with two notable challenges: a) large genome size of garlic, b) strictly asexual life cycle of the cultivated crop. According to the flow cell analysis, the size of diploid (2n = 16) garlic nuclear genome is estimated to be 15.9 Gbp, which is 32 times larger than the rice genome [3], thus posing a challenge to completely sequence the garlic genome. Recent studies accomplished the de novo assembly of garlic transcriptome and generated more than 120,000 transcripts (average length less than 600 bp) [2, 4, 5]. However, their size indicates the presence of a large number of incomplete and redundant transcripts. Hence, these sequence fragments from transcriptome are still rudimentary to be of any use in characterizing the garlic traits. Further, there is no known commercial cultivar or landrace that produces fertile flowers or seeds in garlic, presenting a challenge in the development of a population for genetic studies. Continuous research efforts in restoring fertility have made the self- and cross-pollination within and between some garlic genotypes a reality [6], yet efficient production of garlic seed is in its infancy. So far in garlic, only two low-density genetic maps were generated from a population of 53 plants [7], and none of the quantitative trait loci (QTLs) could be identified.

Genome-wide association study (GWAS) provides a powerful tool for identifying the genes underlying complex traits [8–10]. However, to identify the candidate gene involved in a trait, a reference genome of the species under study is essential for GWAS, which markedly restricts the application of this technology in those species whose genomes remain uncharacterized. Unlike the genome analysis, transcriptome analysis by next-generation sequencing is rapid, inexpensive, and unconstrained by the genomic complexity [11], and its application in association analysis of traits has extended the genetic association studies to a broad range of species, especially to the complex polyploid species [12, 13]. Genome sequence of a progenitor or related species is, however, still required in the studies of associative transcriptomics.

Recently, our team developed a transcriptome-referenced association study (TRAS) that integrated association studies and correlation analysis between gene expression and phenotype to identify the genes potentially involved in a trait, independent of the reference genome [14]. Based on this approach, our team dissected the genetic architecture of three clove shape traits of garlic bulbs and identified 42, 27, and 10 SNPs (from 27, 18, 8 transcripts, respectively) were associated with clove length, width, and thickness, respectively. Of these transcripts, 22 also showed a significant correlation with the corresponding trait in the expression level and were deemed as clove shape-related transcripts. Besides, the eQTL analysis found that 13 of the 22 clove shape-related transcripts exhibited a potential interaction [14]. The characterization of the genetic basis of clove shape traits confirms the feasibility of the TRAS approach.

Axillary expanding buds seen in garlic are the major parts consumed in this crop and such abnormal structures are rare amongst vascular plants. Chasing for high yields is an imperative goal in garlic breeding. The bulbs’ high-yield breeding is, however, markedly limited owing to the unknown genetic basis of this trait. Therefore, in this study, three yield traits such as the bulb weight (BW), bulb diameter (BD), and the number of garlic cloves (CN), were included for the genetic analysis by TRAS in garlic. The characterization of the genetic basis of bulb-yield traits will not only be helpful for the high-yield breeding of garlic bulbs, but also provide a basis for elucidating the mechanism of bulb organ formation in the future.

## Methods

### Experimental population and phenotypic measurements

A population comprising of 102 garlic landraces reported in our previous study [14] were grown in the experimental farm (28°11′49″ N, 112°58′42″ E) of the Institute of Bast Fiber Crops (IBFC), Chinese Academy of Agricultural Sciences (CAAS), Changsha, China, during the growing season in 2015 and 2016. Replicates (two) were grown in a randomized complete block design. For each landrace, thirty-six garlic cloves were planted into a three-row plot, with a distance of 20 cm between the rows and 10 cm between the plants, in each replicate. Thirty plants from the mid-row of each plot were harvested individually when their bulbs were ripe. After air-drying, the bulbs were individually weighed, their diameter was measured using vernier calipers and clove numbers were counted.

### Association analysis

In a previous study, we carried out single-molecule long-read sequencing for a landrace from Yangxi (China) to obtain a high-quality reference transcriptome of 36,321 transcripts (GenBank accession number GFYZ00000000). Illumina sequencing of all 102 landraces (GEO accession number GSE102157), using the RNAs of the developing bulbs was also done [14]. Gene expression levels (GE) of each transcript was estimated and 19,912 high-quality SNPs were identified [14]. Based on the polymorphisms observed, a transcriptome-wide analysis was carried out to detect the loci associated with the bulb-yield traits (BW, BD, and CN). All 19,912 high-quality SNPs detected were further analyzed. To minimize false positives and increase the statistical significance we used mixed linear model program GEMMA v.0.94.1 [15] for the association analysis. The first three principal component analysis values derived from all 19,912 SNPs were used as fixed effects in the mixed model to correct for stratification [16]. The random effect was estimated from groups clustered based on kinship among all accessions, derived from all SNPs. The *P*-value threshold for association loci was set to 2.5 × 10^− 6^ at 5% significance, which was calculated by Bonferroni correction based on the effective number of independent markers [17]. Pearson’s correlation between the expression of the transcript with the associated SNP and the trait value were analyzed in the population, to validate the association between the identified locus and the trait. A significant correlation was achieved at *P* < 0.05. A transcript which associated with a trait at the level of both sequence and expression is considered as a trait-related transcript.

### eQTL analysis

eQTL analysis was used to identify genetic variants that affect the gene expression. This has been frequently applied to detect the interactions and determine the regulatory relationship between genes. To detect the relationship among associated transcripts, 102 landraces were subjected to eQTL analysis using a mixed linear model [15]. Briefly, the 19,912 SNPs were defined as markers, and the expression of associated transcripts were considered as phenotypes. The *P*-value threshold was set to 2.5 × 10^− 6^ at 5% significance. If a SNP showed significant association with the expression of one transcript, this SNP was defined as eQTL, and corresponding transcript into which associated SNP fell was defined as eQTL-located-transcript. We hypothesized that the two transcripts (A and B) have interrelation when they meet the demands as follows: 1) there is a SNP in the transcript B, and it shows a significant association with the expression of transcript A, i.e., the transcript B is the eQTL-located-transcript of A, 2) both A and B transcripts are involved in the same trait by association analysis with this trait, 3) the expression of A transcript is correlated with that of B transcript (*P* < 0.05) [14].

### Identification of co-expressed modules correlating with the bulb-yield traits

Prior co-expression studies analyzing the transcripts from 102 genotypes was conducted using weighted gene co-expression network analysis (WGCNA) software which detected 36,321 transcripts that were assigned to 46 co-expression modules [14]. To identify if these modules allied with yield traits of garlic bulbs, the eigenvalues of each module was used to analyze its association with the traits based on Pearson’s correlation coefficients and a *P-*value less than 0.05 was considered significant.

### Prediction of long non-coding RNA (lncRNA) function

Co-location and co-expression methods were used to determine the *lncRNA* function [18]. Due to the unavailability of complete garlic genome sequence, only the latter method was employed. Briefly, the transcripts co-expressing with lncRNA were identified by WGCNA, and GO enrichment analysis was carried out to predict the function of the lncRNA. Adjusted *P-* values were calculated using the false discovery rate to determine the significance of the enrichment analysis [19]. Co-expression networks were constructed using Cytoscape [20].

## Results

### Population variation

**Fig. 1:**
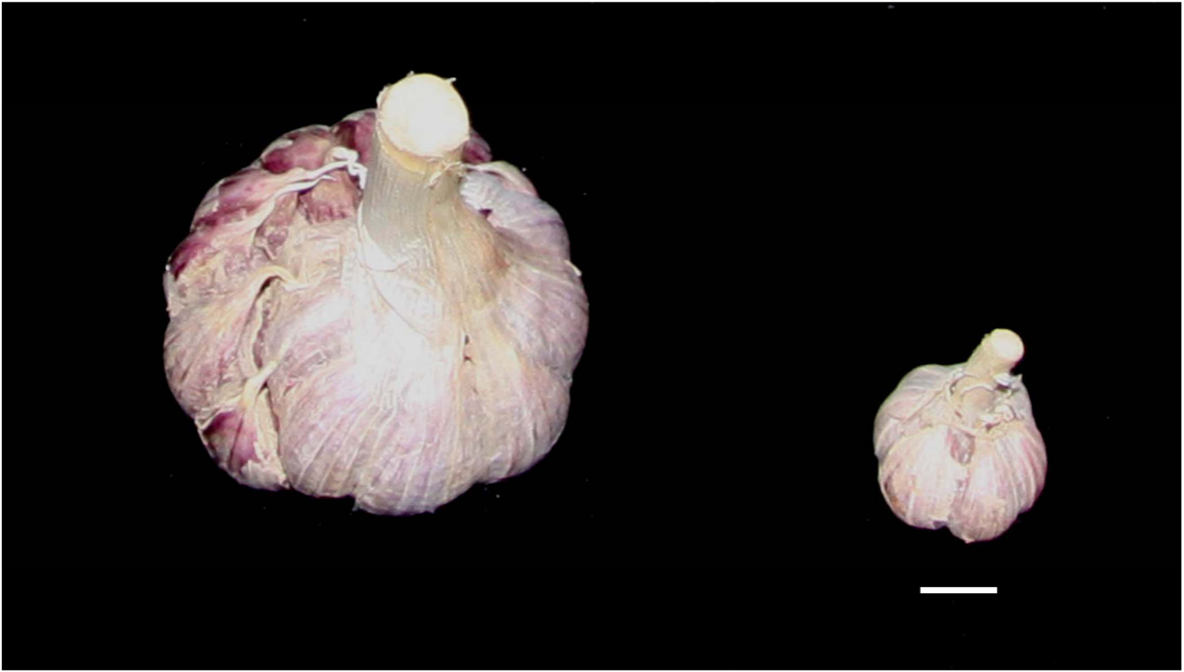
Difference in the bulb size observed between two landraces collected from Chengdu of China (left) and Bengal (right). The scale bar is 10 cm

Variations were observed in the BD, BW, and CN traits in the population. The landrace collected from Chengdu (China) has the largest bulbs (40.8 g in weight and 56.8 mm in diameter), whereas the landrace from Bengal (Asia) showed the smallest bulbs with only 2.4 g in weight and 22.3 mm in diameter (Table 1, Fig. 1). The number of garlic cloves ranged from 5.8 to 35.3, with 6.1-fold differences among the 102 landraces (Table 1). The average trait values of BW, BD, and CN were 21.0 g, 41.2 mm, and 12.7, respectively, in the experiment population (Table 1). Significant positive correlations were observed among the three traits (*P* < 0.05), with a correlation coefficient of 0.92 between BW and BD, 0.36 between BD and CN, and 0.23 between BW and CN.

**Table 1:** Summary of population bulbs-yield traits phenotype and associated loci

Trait	Phenotype		Associated loci	
	Range	Mean ± SD	SNP number	Number of SNP-located-transcript
BW (g)	2.4–40.8	21.0 ± 7.4	25	16
BD (mm)	22.3–56.8	41.2 ± 6.7	2	2
CN	5.8–35.3	12.7 ± 4.9	30	27

### Association analysis of three traits

**Fig. 2:**
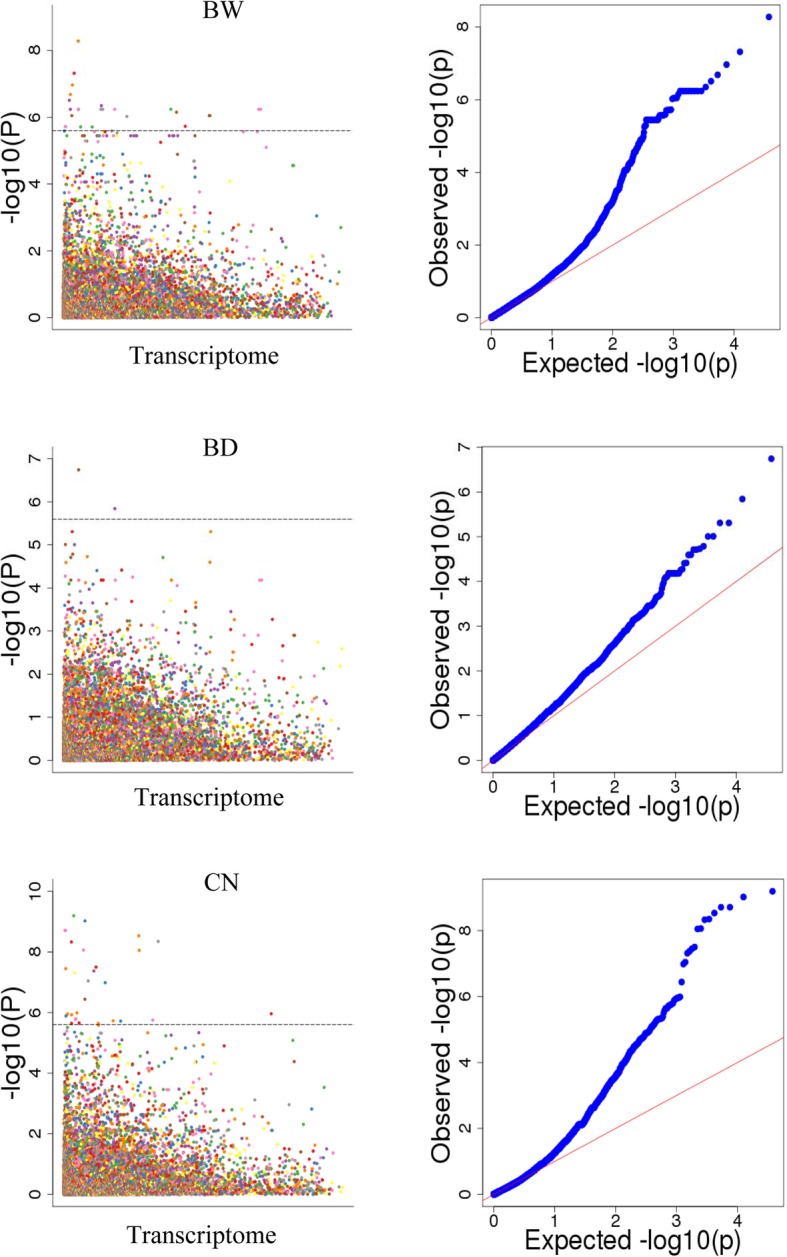
Manhattan and quantile-quantile plots result from the transcriptome-based association study (TRAS) data for the bulb weight (BW), bulb diameter (BD), and the number of garlic cloves (CN), respectively

Association analysis between 19,912 SNPs and three yield traits was carried out and 25, 2, and 30 SNPs were detected that associated with BW, BD, and CN traits, respectively (*P* < 2.5 × 10^− 6^; Fig. 2, Table 1 and Additional file 1: Table S1). Among these SNPs, the 25 BW-associated SNPs fell into 16 transcripts, and 30 CN-associated SNPs were located on 27 transcripts; 2 BD-associated SNPs were derived from two transcripts, *ASTG33285* and *ASTG155*, respectively (Table 1). Thus, a total of 16, 2, and 27 genetic loci associating with the BW, BD, and CN traits were identified finally. Interestingly, the region near *ASTG155* is a pleiotropic locus for both BD and BW traits.

### Identification of transcripts related to bulb-yield traits

Association analysis identifies the genome region with markers present in the linkage disequilibrium (LD) for the loci controlling the trait, and this LD region probably harbors several genes, resulting in a challenge to determine the candidate gene for the target traits. Therefore, to identify the transcripts related to three bulb-yield traits, the correlation analysis between the expression of 16, 2, and 27 transcripts into which associated SNPs localized and the corresponding trait phenotype was carried out. The results revealed a significant correlation in the expression of transcripts 2 and 15 with BW and CN, respectively; however, none of the transcripts showed a correlation with BD (*P* < 0.05; Additional file 1: Table S2). Expression levels of transcripts 1 and 14 exhibited a significant positive correlation with BW and CN respectively, suggesting that they positively control the corresponding traits. In contrast, the two residual transcripts, *ASTG1209* and *ASTG29985*, were found to negatively regulate BW and CN, respectively. As both the sequence and expression levels are associated with the traits, we conclude that the transcripts 2 and 15 are related to BW and CN, separately, i.e., they were yield-related transcripts (Table 2).

**Table 2:** Candidate transcripts identified for bulb-yield traits

Traits	Candidate transcript	CEM ^a^	*P* value ^b^		RD ^c^	Annotation
			Genotype	Expression		
BW	*ASTG1209*	M16	8.9 × 10^−7^	4.2 × 10^−2^	–	alpha-1,4 glucan phosphorylase L-1 isozyme
	*ASTG34835*	M3	1.9 × 10^−6^	2.0 × 10^−2^	+	lncRNA
CN	*ASTG1099*	M41	2.2 × 10^−6^	1.4 × 10^−2^	+	uncharacterized protein
	*ASTG13201*	M15	2.0 × 10^−6^	3.1 × 10^−5^	+	homeobox-leucine zipper protein
	*ASTG208*	M43	1.0 × 10^−6^	1.3 × 10^−9^	+	lncRNA
	*ASTG2382*	M43	1.6 × 10^−6^	4.5 × 10^−5^	+	Cystathionine beta-lyase
	*ASTG28883*	M43	1.0 × 10^−7^	1.4 × 10^−2^	+	phospholipase
	*ASTG29757*	M43	1.1 × 10^−6^	8.1 × 10^−7^	+	cytokinin riboside 5′-monophosphate phosphoribohydrolase
	*ASTG29985*	M8	1.9 × 10^−6^	4.0 × 10^−3^	–	uncharacterized protein
	*ASTG3011*	M43	4.9 × 10^−8^	1.1 × 10^−9^	+	lncRNA
	*ASTG34481*	M43	2.9 × 10^−9^	3.5 × 10^− 9^	+	lncRNA
	*ASTG34606*	M43	1.8 × 10^−6^	7.0 × 10^−10^	+	uncharacterized protein
	*ASTG35290*	M43	3.6 × 10^−7^	2.1 × 10^−7^	+	lncRNA
	*ASTG36040*	M43	3.2 × 10^−8^	1.8 × 10^−4^	+	lncRNA
	*ASTG36172*	M9	2.3 × 10^−6^	3.2 × 10^−4^	+	U11/U12 small nuclear ribonucleoprotein
	*ASTG639*	M41	4.7 × 10^−9^	9.5 × 10^−3^	+	UBX domain-containing protein
	*ASTG9975*	M15	4.5 × 10^−9^	1.3 × 10^−3^	+	ankyrin repeat and SAM domain-containing protein

^a^CEM, co-expression modules; ^b^ The genotype *P* value indicates the associated significance between SNPs of candidate transcript and traits, and the expression *P* value represents the significance of correlation between the expression of candidate transcript and traits; ^c^ RD is an abbreviation of regulation direction, and “+” and “-” indicates that the candidate transcript positively and negatively regulates the trait, respectively

### Characterization of the bulbs yield-related transcripts

Forty six co-expression modules (CEMs) were identified from the referenced transcriptome by WGCNA software. Analysis of the CEMs harboring yield-related transcripts revealed 17 bulb yield-related transcripts that were assigned into seven CEMs (Table 2). Interestingly, 9 of 15 CN-related transcripts belong to the CEM M43. Correlation analysis revealed that a significant positive correlation between the expression of M43 and CN trait (r = 0.452, *P* = 5.1 × 10^− 7^; Additional file 1: Table S3). These results indicated that M43 is important in the regulation of CN traits.

**Fig. 3:**
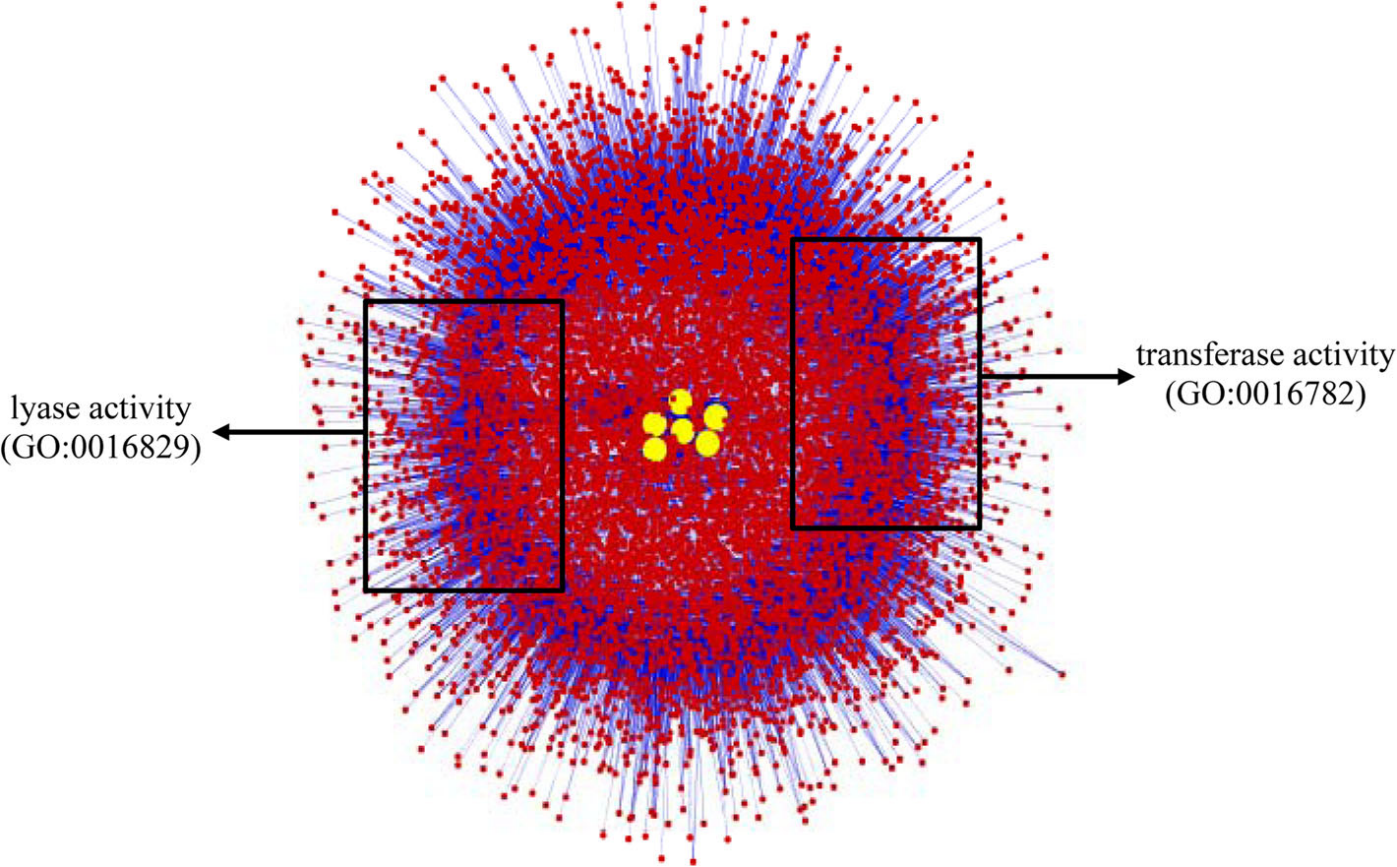
Visualization of a network consisting of eight long non-coding RNAs (*lncRNA*s) related to bulb-yield characteristics and their co-expressed transcripts. Yellow and red dots represent *lncRNAs* identified as candidate transcripts and their co-expressed transcripts, respectively

Among the 17 yield-related transcripts of garlic bulbs, 11 were annotated as protein-encoding transcripts that are involved in carbohydrate metabolism (*ASTG1209*), transcription factor function (*ASTG13201*), enzyme activity (*ASTG2382* and *ASTG28883*), cytokinin activity (*ASTG29757*), U11/U12 small nuclear ribonucleoprotein function (*ASTG36172*), protein degradation (*ASTG639*), SAM domain-containing protein function (*ASTG9975*), and three transcripts encoded for uncharacterized proteins (Table 2). Six residual transcripts were identified as lncRNAs. Further, the potential function of these six lncRNAs was predicted by co-expression method. The results identified approximately 6000–8000 transcripts that were co-expressed with the lncRNAs (Fig. 3), and they were enriched into 10 GO terms, including lyase activity (GO:0016829) and transferase activity (GO:0016782; Fig. 3, Additional file 1: Table S4).

### Detecting interrelation among bulbs yield-related transcripts

The eQTL analysis for 17 yield-related transcripts of garlic bulbs was performed to detect their interrelation, resulting in a total of 642 expression-associated SNPs involved in 446 transcripts (*P* < 2.5 × 10^− 6^; Additional file 1: Table S5). Interestingly, there were a variable number of SNPs associated with the expression of these 17 transcripts. More than 50 SNPs were identified to be associated with the expression of *ASTG2382*, *ASTG3011*, *ASTG9975*, and *ASTG35290* (involved in 250, 55, 69, and 108 transcripts, respectively), indicating that the expression of these four transcripts are modulated by many genetic loci and that they probably function downstream of the pathway for trait regulation. No SNPs were identified to be associated with the expression of *ASTG639*, *ASTG1099*, *ASTG29985*, and *ASTG36172* suggesting that the expression of these transcript is rarely regulated by other genetic loci and that they possibly function upstream from the pathway involved in the trait regulation.

**Fig. 4:**
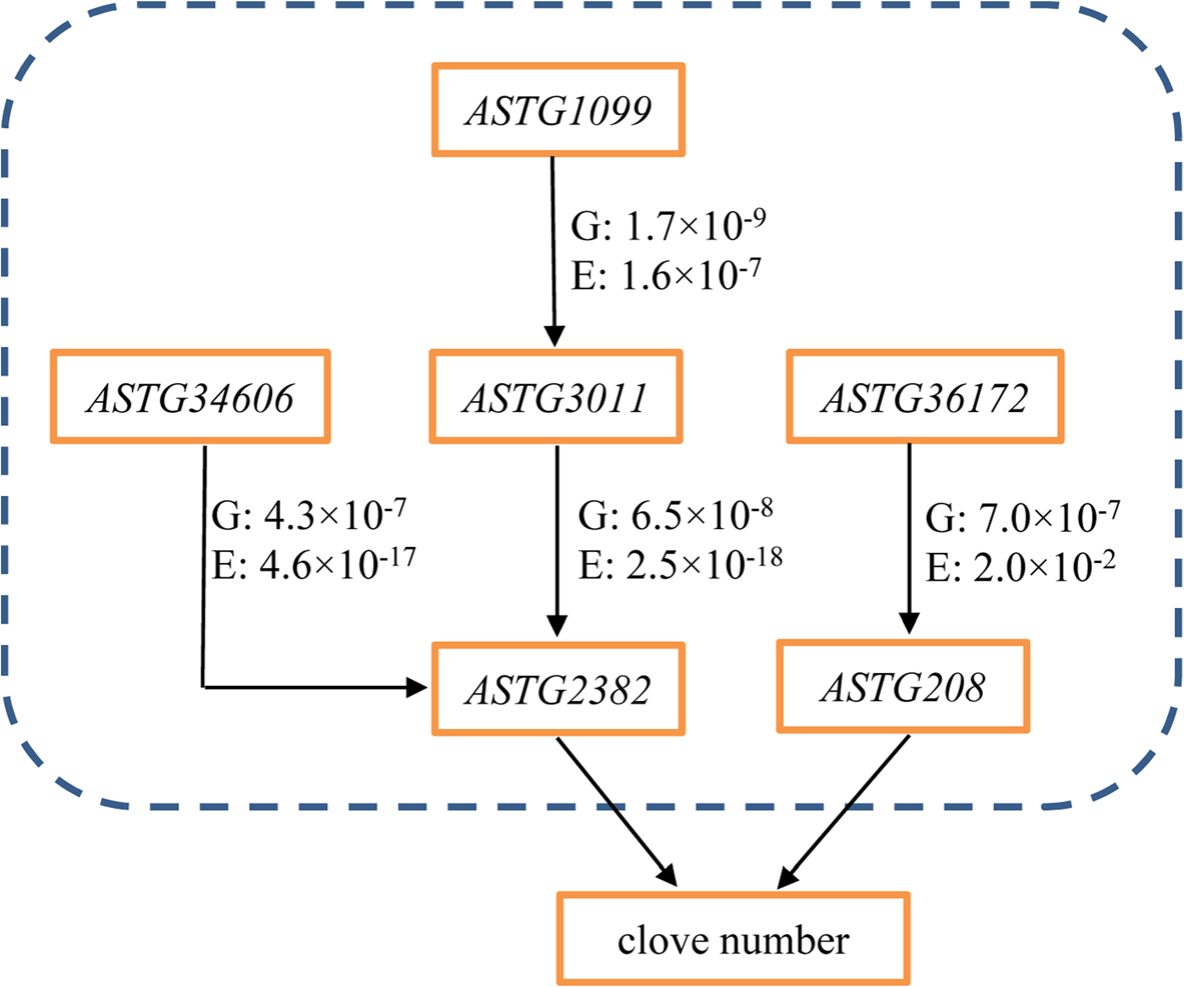
Potential interaction network involved in the regulation of the number of garlic cloves. “G” indicates the *P*-value in eQTL analysis, and “E” represents the *P*-value in the correlation analysis between the expression levels of two transcripts

We hypothesized that the two transcripts (A and B) have interrelation when they meet the following criteria: 1) the expression-associated SNP (eQTL) of A transcript is located on B transcript, 2) both A and B transcripts are related to the same trait, 3) there is a significant correlation between the expression of two transcripts [11]. Finally, we found that, among the 446 eQTL-located-transcripts, four were associated with the CN trait, including the *ASTG36172* (eQTL-located-transcript of *ASTG208*), *ASTG3011* (eQTL-located-transcript of *ASTG2382*), *ASTG34606* (eQTL-located-transcript of *ASTG2382*), *ASTG1099* (eQTL-located-transcript of *ASTG3011*). Furthermore, significant positive correlation between the expression level of these three transcripts and that of the corresponding eQTL-located-transcript was observed (*P* < 0.05). As all the three transcripts (*ASTG208*, *ASTG2382*, and *ASTG3011*) and their eQTL-located-transcripts (*ASTG36172*, *ASTG3011*, *ASTG34606*, and *ASTG1099*) are related to CN traits, and they had a significant correlation in expression, we deduced that there was an interrelation between these transcripts and their corresponding eQTL-located-transcript. Based on the relationship between these seven CN-associated transcripts, two probable pathways involved in CN trait were identified: one, in which *ASTG36172* influences the expression of *ASTG208*, and another, in which *ASTG1099* influences the expression of *ASTG3011*, thereby *ASTG3011* together with *ASTG34606* modulate the expression of *ASTG2382*, finally to determine CN (Fig. 4).

## Discussion

BD, BW, and CN are three most valuable traits in garlic production. Owing to the large genome size and a strictly asexual life cycle of the cultivar, the genetic and molecular basis of these three traits are poorly understood. In this study, the genetic architecture of three of the bulb-yield traits were first dissected by association mapping, resulting in a total of 16, 2, and 27 genetic loci identified for BW, BD, and CN traits, respectively. The identification of these loci will provide basis for cloning the genes involved in corresponding trait.

It is notable that a significant positive correlation amongst these three bulb-yield traits, especially between BD and BW was observed. Previous studies indicated that the genetic basis of correlation between traits was due to the pleiotropic loci associated with correlated traits [21, 22]. However, it is puzzling that only one pleiotropic locus has been identified for BD and BW. Interestingly, when using the (1/N) *P-* value threshold (5 × 10^− 5^) in association analysis, 12, 60, 101 suggestive loci were identified for BD, BW, and CN, respectively (Additional file 1: Table S6). Many of these were pleiotropic, including 8 and 2 of 12 suggesting the BD-associated loci with additional effect on BW and CN, respectively. Additionally, 2 loci exhibited pleiotropism for BW and CN. However, the association between suggestive loci and corresponding traits was not significant, and their pleiotropic effect needs to be further confirmed in future study.

This study thus identified 2 and 15 transcripts related to BW and CN, respectively. Among the two BW-related transcripts, one (*ASTG1209*) encodes an alpha-1,4 glucan phosphorylase. It is notable that carbohydrates are the main component of garlic bulbs [23], and its metabolism contributes to the bulb development. As a gene involved in carbohydrate metabolism, *ASTG1209* probably plays a key role in controlling BW traits. Interestingly, previous study revealed that *ASTG1209* was involved in the regulation of length and width of garlic clove [14]. Therefore, *ASTG1209* is a pleiotropic transcript dictating the clove shape and bulb yield.

It is known that garlic cloves are abnormal axillary buds, and thus, the initiation and development of axillary meristems is important in determining the number of cloves. In *Arabidopsis*, hormones have important roles in modulating initiation and development of axillary buds, *ex:* abscisic acid inhibits the growth of buds, and cytokinin promotes the initiation of buds [24, 25]. Hormone signaling is regulated by transcription factors. At least three homeodomain leucine zipper (HD-ZIP) genes were found to increase the abscisic acid accumulation, and trigger hormone response, thus causing suppression of bud development [26]. In this study, transcripts encoding cytokinin-activity and a HD-ZIP transcription factor were associated with CN in both sequence and gene expression, confirming the potential role of hormones in the regulation of CN.

Several genes associated with protein degradation, include *MAX2*, *UCH1*, and *UCH2*, which play an essential role in the regulation of formation and growth of axillary buds [27, 28]. UBX domain-containing protein has been shown to participate broadly in the regulation of protein degradation [29–31]. In the present study, a garlic UBX domain-containing protein-encoding transcript, *ASTG639*, was found to be related to the CN. Therefore, protein degradation is possibly involved in the regulation of the CN.

SAM domain-containing proteins carry out diverse important functions in eukaryotes, serving as protein–protein interaction motifs and binding targets to a variety of substrates including RNA and lipids [32]. *Arabidopsis LEAFY* is a transcription factor containing SAM domain involved in cell differentiation, flower formation, and meristem identity [33]. In this study, a SAM domain-containing protein-encoding transcript was found to be linked to the regulation of CN.

Further, two enzyme-encoding transcripts were found to be associated with the CN. Moreover, all 5 CN-related *lncRNAs* were potentially involved in the function of lyase and/or transferase activity. These results suggest that metabolic enzymes play a role in the control of the CN. However, three candidate protein-coding transcripts were uncharacterized; indicating their role in the regulation of the CN to provide new insights into the molecular basis of the CN formation.

To conclude, a total of 56 SNPs from 44 loci associated with three of the bulb-yield traits were detected in garlic. Furthermore, 17 transcripts related to bulbs yield were identified, of which 7 had an interrelation. Six transcripts were *lnc*RNAs, and the others were proteins involved majorly in carbohydrate metabolism, transcription regulation, cytokinin activity, etc. This study provides new insights into the genetic basis of garlic yield traits, and the associated SNPs/transcripts of traits identified herein provide a basis for improving the bulb-yield in garlic breeding.

## Additional file

Additional file 1: **Table S1** Associated SNPs with three garlic bulb-yield traits (*P* < 2.5 × 10^− 6^). **Table S2** Correlation between the expression of the associated transcripts and corresponding bulb-yield traits (*P* < 2.5 × 10^− 6^). **Table S3** Co-expression modules significantly correlated with the bulb-yield traits (*P* < 0.05). **Table S4** Enriched Gene Ontology terms for transcripts co-expressed with long non-coding RNAs (*Q* value < 0.05). **Table S5** Single nucleotide polymorphisms (SNPs) associated with the expression of the 17 transcripts. **Table S6** Suggestive loci associated with three garlic bulb-yield traits (*P* < 5 × 10^− 5^) (PDF 781 kb)

## Data Availability

All data generated or analysed during this study are included in this published article [and its supplementary information files].

## Abbreviations

BD: Bulb diameter
BW: Bulb weight
CEM: Co-expression modules
CN: The number of garlic cloves
eQTL: Expression quantitative trait loci
GE: Gene expression
GWAS: Genome-wide association study
HD-ZIP: Homeodomain leucine zipper
lncRNA: Long non-coding RNA
SNP: Single nucleotide polymorphism
TRAS: Transcriptome-referenced association study
