# Supplementary material for: Transcriptome-wide association study and eQTL analysis to assess the genetic basis of bulb-yield traits in garlic (Allium sativum)
Source: BMC Genomics. 2019 Aug 17;20:657. doi: 10.1186/s12864-019-6025-2 (PMC6698038; doi:10.1186/s12864-019-6025-2)
Supplement: Supplementary file 1 — Table S1 Associated SNPs with three garlic bulb-yield traits (P < 2.5 × 10− 6). Table S2 Correlation between the expression of the associated transcripts and corresponding bulb-yield traits (P < 2.5 × 10− 6). Table S3 Co-expression modules significantly correlated with the bulb-yield traits (P < 0.05). Table S4 Enriched Gene Ontology terms for transcripts co-expressed with long non-coding RNAs (Q value < 0.05). Table S5 Single nucleotide polymorphisms (SNPs) associated with the expression of the 17 transcripts. Table S6 Suggestive loci associated with three garlic bulb-yield traits (P < 5 × 10− 5) (PDF 781 kb) [file 12864_2019_6025_MOESM1_ESM.pdf]

Table S1 Associated SNPs with three garlic bulb-yield traits ($P < 2.5\times10^{-6}$)

Traits	Transcript involved	SNP position in transcript	REF	ALT	maf	P value
BW	ASTG10870	1690	A	G	0.191	1.86×10^{-6}
	ASTG1209	2022	A	G	0.13	8.88×10^{-7}
	ASTG1209	2033	A	G	0.132	8.88×10^{-7}
	ASTG155	99	G	A	0.097	2.06×10^{-7}
	ASTG155	127	C	T	0.094	1.08×10^{-7}
	ASTG155	208	A	G	0.079	5.3×10^{-9}
	ASTG16546	881	A	T	0.077	9.42×10^{-7}
	ASTG1912	528	C	T	0.246	4.44×10^{-7}
	ASTG22711	84	A	G	0.167	3.08×10^{-7}
	ASTG29913	1491	G	A	0.219	5.73×10^{-7}
	ASTG33002	151	A	G	0.178	4.79×10^{-8}
	ASTG33394	210	T	G	0.215	5.73×10^{-7}
	ASTG33450	521	T	C	0.242	5.73×10^{-7}
	ASTG33450	546	A	T	0.226	5.73×10^{-7}
	ASTG34285	1569	A	T	0.375	7.0×10^{-7}
	ASTG34835	241	A	G	0.282	1.94×10^{-6}
	ASTG34835	402	G	A	0.282	1.94×10^{-6}
	ASTG35417	28	T	C	0.144	1.9×10^{-6}
	ASTG35417	35	C	A	0.144	1.9×10^{-6}
	ASTG35854	99	T	A	0.233	5.73×10^{-7}
	ASTG4710	119	G	T	0.069	8.88×10^{-7}
	ASTG830	212	A	G	0.215	5.73×10^{-7}
	ASTG830	713	C	A	0.215	5.73×10^{-7}
	ASTG830	2709	G	T	0.215	5.73×10^{-7}
	ASTG830	2736	G	A	0.215	5.73×10^{-7}
BD	ASTG155	208	A	G	0.079	1.81×10^{-7}
	ASTG33285	708	C	T	0.086	1.44×10^{-6}
CN	ASTG1099	213	A	G	0.073	2.21×10^{-6}
	ASTG1118	31	A	G	0.078	1.3×10^{-6}
	ASTG13201	18	T	A	0.086	2.01×10^{-6}
	ASTG1525	415	C	T	0.062	4.25×10^{-8}
	ASTG207	138	G	T	0.063	6.42×10^{-10}
	ASTG208	183	G	A	0.063	1.03×10^{-6}
	ASTG2382	168	T	A	0.068	1.6×10^{-6}
	ASTG25083	2863	T	C	0.066	1.09×10^{-6}
	ASTG28883	572	T	C	0.073	1.03×10^{-7}
	ASTG29757	52	T	G	0.05	1.11×10^{-6}

ASTG29985	682	C	T	0.13	1.87×10^{-6}
ASTG3011	152	T	A	0.098	4.87×10^{-8}
ASTG34481	1041	C	A	0.196	8.91×10^{-9}
ASTG34481	1035	T	A	0.188	2.94×10^{-9}
ASTG34606	1228	T	C	0.089	1.77×10^{-6}
ASTG35290	292	C	A	0.083	3.63×10^{-7}
ASTG35328	16	T	C	0.058	1.95×10^{-9}
ASTG35328	25	G	A	0.058	1.95×10^{-9}
ASTG35406	28	T	G	0.115	3.56×10^{-8}
ASTG35422	337	T	C	0.088	9.07×10^{-8}
ASTG35935	109	A	C	0.153	1.19×10^{-6}
ASTG35951	293	G	A	0.118	9.54×10^{-10}
ASTG36040	445	A	G	0.137	3.17×10^{-8}
ASTG36172	476	G	A	0.069	2.29×10^{-6}
ASTG4427	160	T	C	0.17	1.69×10^{-6}
ASTG639	108	A	G	0.087	2.29×10^{-6}
ASTG639	106	C	T	0.09	4.71×10^{-9}
ASTG7586	785	T	A	0.091	1.92×10^{-6}
ASTG977	272	T	C	0.057	8.66×10^{-9}
ASTG9975	1303	A	C	0.058	4.5×10^{-9}

Table S2 Correlation between the expression of the associated transcripts and corresponding bulbs-yield traits ($P < 2.5\times10^{-6}$)

Transcripts	Traits	P value	Correlation coefficient	Transcripts	Traits	P value	Correlation coefficient
ASTG33285	BD	0.627	0.05	ASTG208*	CN	1.28×10^{-9}	0.60
ASTG155	BD	0.597	−0.06	ASTG2382*	CN	4.52×10^{-5}	0.43
ASTG10870	BW	0.327	0.11	ASTG25083	CN	0.745	0.04
ASTG1209*	BW	0.042	−0.23	ASTG28883*	CN	0.014	0.27
ASTG155	BW	0.519	−0.07	ASTG29757*	CN	8.11×10^{-7}	0.51
ASTG16546	BW	0.523	0.07	ASTG29985*	CN	4.0×10^{-3}	−0.31
ASTG1912	BW	0.229	0.13	ASTG3011*	CN	1.06×10^{-9}	0.61
ASTG22711	BW	0.277	−0.12	ASTG34481*	CN	3.54×10^{-9}	0.59
ASTG29913	BW	0.981	0.00	ASTG34606*	CN	6.95×10^{-10}	0.61
ASTG33002	BW	0.317	0.11	ASTG35290*	CN	2.1×10^{-7}	0.53
ASTG33394	BW	0.519	0.07	ASTG35328	CN	0.256	−0.13
ASTG33450	BW	0.519	−0.07	ASTG35406	CN	0.889	0.02
ASTG34285	BW	0.289	0.12	ASTG35422	CN	0.066	0.20
ASTG34835*	BW	0.020	0.26	ASTG35935	CN	0.093	0.18
ASTG35417	BW	0.910	0.01	ASTG35951	CN	0.966	0.00
ASTG35854	BW	0.708	−0.04	ASTG36040*	CN	1.77×10^{-4}	0.40
ASTG4710	BW	0.071	0.20	ASTG36172*	CN	3.2×10^{-4}	0.38
ASTG830	BW	0.075	0.20	ASTG4427	CN	0.052	−0.21
ASTG1099*	CN	0.014	0.27	ASTG639*	CN	9.52×10^{-3}	0.28
ASTG1118	CN	0.283	0.12	ASTG7586	CN	0.371	−0.10
ASTG13201*	CN	3.07×10^{-5}	0.44	ASTG977	CN	0.067	0.20
ASTG1525	CN	0.952	0.01	ASTG9975*	CN	1.26×10^{-3}	0.35
ASTG207	CN	0.245	0.13				

Table S3 Co-expression modules significantly correlated with the bulbs-yield traits (P < 0.05).

CEMs	CN		BD		BW	
	Correlated coefficient	*P* value	Correlated coefficient	*P* value	Correlated coefficient	*P* value
M3	0.197	0.037			0.251	0.007
M4	－0.250	0.008			－0.228	0.015
M6	－0.283	0.002			－0.246	0.009
M8	－0.317	0.001			－0.245	0.009
M9	0.216	0.022			0.209	0.027
M15	0.241	0.010			0.265	0.005
M16	－0.297	0.001			－0.191	0.043
M40	－0.324	4.6×10^{-4}			－0.236	0.012
M41	0.197	0.037	0.483	6.1×10^{-8}		
M43	0.452	5.1×10^{-7}			0.599	2.5×10^{-12}

Table S4 Enriched Gene Ontology terms for transcripts co-expressed with long non-coding RNAs (Q value < 0.05)

lncRNA	GO_accession	Description	Term_type	P value	Q value
ASTG208	GO:0015074	DNA integration	biological_process	2.75E-14	1.33E-10
	GO:0016846	carbon-sulfur lyase activity	molecular_function	1.88E-11	4.56E-08
	GO:0016829	lyase activity	molecular_function	7.85E-06	0.012707
	GO:0016782	transferase activity, transferring sulfur-containing groups	molecular_function	1.66E-05	0.020142
ASTG3011	GO:0015074	DNA integration	biological_process	4.21E-14	2.04E-10
	GO:0016846	carbon-sulfur lyase activity	molecular_function	8.41E-11	2.04E-07
	GO:0016829	lyase activity	molecular_function	4.51E-06	0.0072906
	GO:0016782	transferase activity, transferring sulfur-containing groups	molecular_function	1.93E-05	0.023388
ASTG34481	GO:0015074	DNA integration	biological_process	1.08E-14	5.24E-11
	GO:0016846	carbon-sulfur lyase activity	molecular_function	2.09E-12	5.07E-09
	GO:0016829	lyase activity	molecular_function	9.16E-06	0.014447
	GO:0016782	transferase activity, transferring sulfur-containing groups	molecular_function	1.19E-05	0.014447
ASTG34835	GO:0015074	DNA integration	biological_process	1.41E-06	0.0068295
	GO:0006364	rRNA processing	biological_process	7.23E-06	0.0094831
	GO:0016072	rRNA metabolic process	biological_process	7.23E-06	0.0094831
	GO:0008152	metabolic process	biological_process	7.82E-06	0.0094831
	GO:0006396	RNA processing	biological_process	1.36E-05	0.013248
ASTG35290	GO:0015074	DNA integration	biological_process	3.05E-13	1.48E-09
	GO:0016846	carbon-sulfur lyase activity	molecular_function	8.42E-13	2.04E-09
	GO:0016829	lyase activity	molecular_function	4.91E-06	0.0079364
	GO:0016782	transferase activity, transferring sulfur-containing groups	molecular_function	1.27E-05	0.015351
	GO:0030170	pyridoxal phosphate binding	molecular_function	1.78E-05	0.01723
ASTG36040	GO:0015074	DNA integration	biological_process	7.37E-13	3.58E-09
	GO:0016846	carbon-sulfur lyase activity	molecular_function	7.85E-12	1.90E-08
	GO:0016829	lyase activity	molecular_function	3.40E-07	0.00055009
	GO:0016782	transferase activity, transferring sulfur-containing groups	molecular_function	1.77E-05	0.021494

Table S5 Single nucleotide polymorphisms (SNPs) associated with the expression of the 17 transcripts

Candidate transcript	eQTL-located-Transcript	pos	REF	ALT	maf	P value
ASTG208	*ASTG1128*	507	T	C	0.191	9.06E-07
	ASTG1439	975	G	A	0.069	3.95E-07
	ASTG24613	1732	T	C	0.066	5.49E-07
	ASTG253	362	T	C	0.077	2.15E-06
	ASTG253	365	G	T	0.077	2.15E-06
	ASTG3028	1021	G	A	0.242	6.14E-07
	ASTG3277	418	T	A	0.105	1.85E-06
	ASTG33094	854	A	G	0.161	1.47E-06
	ASTG33449	251	T	C	0.109	1.68E-06
	ASTG35660	417	T	C	0.075	8.92E-07
	ASTG35973	841	G	A	0.152	2.14E-06
	ASTG36117	296	A	G	0.066	4.90E-08
	ASTG36172	476	G	A	0.069	7.05E-07
	ASTG44	74	G	T	0.090	6.48E-07
	ASTG7334	28	T	G	0.113	2.30E-06
ASTG639	NS					
ASTG1099	NS					
ASTG1209	*ASTG1100*	701	G	A	0.086	1.50E-09
	ASTG25557	1018	C	T	0.064	2.70E-07
	ASTG25557	889	C	A	0.053	3.74E-08
	ASTG299	631	C	T	0.369	4.15E-08
	ASTG31174	124	A	G	0.143	1.90E-16
	ASTG32934	553	G	T	0.336	7.36E-08
	ASTG34039	663	T	C	0.067	6.64E-07
	ASTG34039	673	A	G	0.067	6.64E-07
	ASTG34779	501	A	G	0.152	1.92E-16
	ASTG35667	470	T	C	0.062	1.17E-08
	ASTG35667	478	C	T	0.065	9.61E-09
	ASTG35908	926	C	T	0.387	1.57E-06
	ASTG35908	1059	T	C	0.101	1.01E-06
	ASTG6706	29	T	C	0.188	1.86E-06
ASTG2382	*Cluster_112_0*	406	C	T	0.084	2.34E-08
	ASTG10	471	G	A	0.098	8.87E-07
	ASTG10	397	G	C	0.098	7.85E-07
	ASTG10	431	C	T	0.097	3.35E-07
	ASTG10825	503	A	G	0.082	1.63E-07
	ASTG10825	523	T	C	0.074	5.81E-08
	ASTG10837	426	C	G	0.065	1.92E-09
	ASTG10870	1016	A	T	0.211	4.28E-08

ASTG11025	31	T	C	0.072	4.83E-09
ASTG1118	31	A	G	0.078	2.34E-06
ASTG1123	452	A	G	0.097	3.33E-22
ASTG1126	370	G	T	0.241	7.51E-08
ASTG1126	481	T	C	0.232	1.99E-10
ASTG11358	3429	C	T	0.069	1.52E-06
ASTG11600	48	T	C	0.063	2.43E-10
ASTG11600	2480	G	A	0.059	5.89E-13
ASTG1209	982	C	G	0.138	8.72E-13
ASTG12152	35	C	T	0.077	1.84E-09
ASTG12265	958	T	C	0.060	1.87E-07
ASTG1227	11	C	G	0.068	2.86E-07
ASTG12548	1482	A	T	0.058	2.64E-07
ASTG12656	844	G	C	0.083	5.16E-08
ASTG12708	104	A	C	0.068	1.72E-06
ASTG13590	793	T	G	0.074	1.35E-06
ASTG13680	589	C	T	0.080	1.78E-08
ASTG13922	1927	T	C	0.127	9.44E-07
ASTG1439	975	G	A	0.069	1.04E-11
ASTG144	222	G	A	0.071	1.89E-13
ASTG1450	785	G	C	0.061	4.03E-23
ASTG1468	611	C	T	0.082	5.57E-08
ASTG14768	91	T	C	0.064	2.17E-09
ASTG15157	22	C	T	0.126	1.41E-07
ASTG15288	703	C	T	0.097	2.51E-08
ASTG16512	52	A	T	0.063	1.22E-06
ASTG1789	683	C	T	0.097	2.37E-09
ASTG18736	1007	T	C	0.052	1.44E-08
ASTG19113	1544	T	G	0.122	5.17E-07
ASTG1938	338	G	C	0.086	1.74E-06
ASTG1955	267	C	T	0.076	3.43E-07
ASTG1957	29	A	G	0.062	1.40E-10
ASTG20404	51	G	A	0.068	6.03E-10
ASTG20404	54	G	A	0.068	6.03E-10
ASTG2106	543	T	C	0.060	5.62E-08
ASTG2106	552	A	C	0.053	3.92E-20
ASTG2240	895	A	T	0.070	1.50E-08
ASTG22698	1099	G	A	0.135	1.42E-09
ASTG22698	1121	C	T	0.134	7.38E-10
ASTG22729	264	A	G	0.074	5.71E-08
ASTG23307	65	C	T	0.063	8.16E-08
ASTG23507	163	C	G	0.125	5.19E-13
ASTG23574	1077	C	T	0.158	8.66E-09
ASTG237	64	T	C	0.065	1.32E-07

ASTG237	124	T	C	0.056	2.51E-09
ASTG23781	20	T	C	0.063	1.50E-06
ASTG23815	22	A	T	0.061	2.16E-21
ASTG2404	361	G	A	0.072	1.22E-08
ASTG2404	182	T	A	0.072	4.46E-12
ASTG241	525	T	G	0.159	3.05E-07
ASTG24228	61	C	A	0.182	7.71E-10
ASTG24228	58	G	C	0.188	6.21E-10
ASTG24228	82	G	A	0.146	5.37E-11
ASTG24228	40	C	T	0.186	2.19E-13
ASTG24767	1271	C	T	0.076	3.35E-07
ASTG25139	205	A	G	0.119	1.94E-08
ASTG26122	196	C	T	0.081	1.99E-06
ASTG2615	61	G	A	0.063	4.57E-10
ASTG2615	59	C	A	0.063	3.24E-10
ASTG26307	20	C	T	0.068	3.42E-09
ASTG26786	1904	T	A	0.073	3.34E-08
ASTG27009	4	A	G	0.061	8.51E-09
ASTG2738	1836	T	C	0.136	1.03E-06
ASTG28223	315	G	T	0.066	8.68E-09
ASTG28223	325	A	T	0.066	6.93E-09
ASTG2863	899	T	A	0.123	3.93E-12
ASTG2883	930	T	G	0.064	9.57E-10
ASTG28884	15	A	G	0.075	1.80E-07
ASTG29101	528	A	C	0.203	2.80E-10
ASTG29101	105	A	T	0.210	4.62E-11
ASTG29101	106	A	T	0.210	4.62E-11
ASTG29101	107	C	T	0.210	4.62E-11
ASTG29101	617	C	T	0.211	9.77E-12
ASTG2912	41	G	A	0.152	1.23E-07
ASTG29361	1686	G	C	0.069	4.14E-07
ASTG2947	34	A	C	0.130	4.76E-08
ASTG29574	508	A	G	0.122	5.00E-07
ASTG2960	59	C	A	0.073	1.45E-09
ASTG29672	409	G	T	0.159	7.59E-07
ASTG3011	152	T	A	0.098	6.54E-08
ASTG30213	479	A	T	0.088	1.42E-06
ASTG3024	400	C	T	0.050	5.90E-08
ASTG30588	901	T	C	0.116	1.39E-11
ASTG30912	70	T	C	0.076	9.34E-07
ASTG30912	92	G	T	0.073	1.38E-07
ASTG30984	1236	G	A	0.213	1.07E-10
ASTG311	778	T	A	0.129	1.88E-08
ASTG31317	672	T	C	0.090	3.54E-08

ASTG31360	57	A	C	0.074	1.30E-07
ASTG31499	67	T	C	0.195	1.72E-06
ASTG31650	425	A	T	0.105	2.47E-06
ASTG31650	723	C	T	0.054	1.46E-09
ASTG31650	382	C	T	0.065	4.63E-12
ASTG31878	357	A	G	0.205	6.17E-07
ASTG31878	360	C	T	0.205	6.17E-07
ASTG31892	270	G	T	0.072	2.33E-08
ASTG31921	37	A	G	0.063	4.41E-09
ASTG32249	1490	G	A	0.122	1.61E-12
ASTG32352	996	A	G	0.089	2.67E-08
ASTG32375	735	C	T	0.079	9.64E-07
ASTG32375	743	G	A	0.078	6.56E-07
ASTG32604	712	T	C	0.112	5.13E-09
ASTG32704	254	T	C	0.058	2.21E-13
ASTG32734	342	G	A	0.364	4.47E-09
ASTG32865	1225	T	G	0.155	7.69E-11
ASTG3303	80	C	A	0.053	8.60E-08
ASTG33094	1084	G	C	0.170	3.51E-07
ASTG33108	117	G	A	0.128	2.86E-09
ASTG33180	73	T	C	0.064	3.05E-07
ASTG33222	30	A	C	0.096	2.32E-08
ASTG33274	1268	G	T	0.051	1.69E-07
ASTG3338	389	A	G	0.185	9.78E-09
ASTG33562	553	A	G	0.245	1.24E-06
ASTG3360	89	C	G	0.115	2.98E-09
ASTG33647	46	A	C	0.083	7.72E-07
ASTG33748	332	A	G	0.052	2.98E-07
ASTG33809	1295	C	G	0.230	6.44E-07
ASTG33845	502	A	C	0.099	5.65E-07
ASTG34007	532	T	C	0.107	1.83E-07
ASTG3403	473	T	G	0.179	2.10E-06
ASTG3403	497	G	A	0.164	1.60E-06
ASTG3403	203	C	T	0.116	7.47E-10
ASTG341	1000	G	T	0.102	1.16E-06
ASTG34126	578	G	A	0.063	5.10E-08
ASTG34133	876	C	A	0.069	1.95E-06
ASTG34133	835	C	T	0.090	4.49E-07
ASTG34157	1656	A	G	0.073	1.18E-09
ASTG34190	1545	T	A	0.092	2.97E-07
ASTG34200	35	A	T	0.074	2.57E-08
ASTG34405	463	T	C	0.104	1.24E-06
ASTG34505	86	A	G	0.067	1.07E-06
ASTG34606	628	T	A	0.088	4.32E-07

ASTG34612	17	T	C	0.070	1.02E-10
ASTG3464	314	T	A	0.079	1.57E-06
ASTG34708	430	A	G	0.063	4.34E-08
ASTG34716	953	C	T	0.102	2.18E-08
ASTG34716	990	T	C	0.118	7.28E-10
ASTG34716	997	A	G	0.118	7.28E-10
ASTG34749	1075	C	G	0.121	4.63E-12
ASTG34765	781	A	C	0.088	3.07E-08
ASTG34781	584	C	T	0.110	7.19E-08
ASTG34781	589	G	A	0.110	7.19E-08
ASTG34894	780	A	C	0.132	8.33E-09
ASTG34950	116	T	A	0.066	9.00E-10
ASTG34950	118	A	G	0.066	9.00E-10
ASTG34974	1368	G	A	0.071	8.09E-08
ASTG34974	1427	A	T	0.073	7.36E-09
ASTG34998	867	T	C	0.119	7.84E-08
ASTG35010	45	G	A	0.063	5.07E-11
ASTG35064	98	A	C	0.122	1.57E-12
ASTG35110	717	A	G	0.066	2.46E-09
ASTG35153	278	T	C	0.079	6.12E-08
ASTG35177	460	C	T	0.051	1.17E-06
ASTG35233	1520	T	C	0.051	2.30E-07
ASTG35233	1054	C	T	0.065	3.27E-14
ASTG35278	122	A	C	0.112	2.48E-08
ASTG35323	363	A	G	0.082	8.30E-08
ASTG35323	374	T	C	0.058	6.89E-09
ASTG35371	343	T	G	0.072	5.88E-11
ASTG35371	352	C	T	0.072	5.88E-11
ASTG35392	749	C	T	0.074	2.26E-08
ASTG354	117	A	G	0.062	1.00E-08
ASTG35422	444	G	A	0.125	8.96E-07
ASTG35435	464	C	T	0.088	6.69E-08
ASTG35475	2179	C	A	0.063	7.96E-13
ASTG35475	2146	G	A	0.061	1.74E-23
ASTG35495	20	C	T	0.064	9.49E-08
ASTG35622	23	T	C	0.111	5.06E-07
ASTG35637	685	T	C	0.062	4.71E-08
ASTG3564	709	G	A	0.195	2.27E-06
ASTG3564	706	G	A	0.211	1.06E-06
ASTG35690	798	A	G	0.200	8.27E-16
ASTG35735	433	C	A	0.133	5.71E-09
ASTG35827	504	T	C	0.081	2.13E-25
ASTG35855	551	C	T	0.122	1.07E-07
ASTG35894	449	T	A	0.220	1.10E-07

ASTG35922	116	T	A	0.115	6.89E-07
ASTG35925	111	A	C	0.059	7.47E-07
ASTG35942	447	G	A	0.108	1.44E-08
ASTG35991	372	A	G	0.065	8.11E-08
ASTG36	79	G	A	0.070	4.03E-07
ASTG36045	300	G	A	0.063	2.10E-10
ASTG36060	299	A	G	0.078	4.98E-07
ASTG36060	371	C	T	0.146	2.15E-09
ASTG36089	411	T	A	0.145	2.88E-07
ASTG36096	511	G	A	0.245	2.39E-07
ASTG36121	81	G	C	0.095	1.61E-06
ASTG36124	177	T	C	0.074	7.11E-07
ASTG36128	296	A	G	0.067	4.42E-12
ASTG36159	44	A	T	0.123	1.01E-06
ASTG36159	41	G	A	0.119	4.94E-07
ASTG369	564	G	T	0.218	6.26E-07
ASTG369	587	G	T	0.220	1.64E-07
ASTG416	945	T	C	0.212	1.37E-10
ASTG425	736	A	C	0.141	2.76E-08
ASTG44	537	A	G	0.086	1.71E-07
ASTG4543	926	G	A	0.066	2.59E-08
ASTG4554	12	A	G	0.066	4.37E-10
ASTG4577	377	A	G	0.105	2.72E-08
ASTG4580	43	T	C	0.098	9.07E-10
ASTG4612	352	G	A	0.074	6.34E-08
ASTG4621	301	C	T	0.071	1.36E-06
ASTG4837	618	C	T	0.092	9.18E-09
ASTG4857	672	C	T	0.115	1.83E-07
ASTG49	158	A	T	0.140	2.00E-06
ASTG5019	1360	A	G	0.205	6.61E-11
ASTG5019	1369	T	G	0.205	6.61E-11
ASTG5048	384	G	A	0.062	1.40E-10
ASTG5132	27	C	G	0.061	2.01E-06
ASTG5183	38	C	T	0.066	2.51E-19
ASTG526	384	T	C	0.082	1.43E-08
ASTG5327	66	G	C	0.066	7.84E-09
ASTG5514	166	G	A	0.078	9.33E-11
ASTG5514	168	T	G	0.078	9.33E-11
ASTG562	188	G	A	0.081	1.15E-12
ASTG587	341	G	A	0.058	9.63E-25
ASTG5903	1923	G	A	0.062	1.40E-10
ASTG6147	1087	G	A	0.051	1.04E-06
ASTG6213	820	A	G	0.068	9.26E-08
ASTG628	1819	T	G	0.106	8.72E-08

	ASTG628	823	C	T	0.075	9.19E-11
	ASTG6326	280	G	A	0.061	6.91E-07
	ASTG6326	277	C	T	0.063	2.77E-07
	ASTG6326	256	A	T	0.127	3.46E-09
	ASTG6557	1782	C	T	0.080	9.87E-07
	ASTG6787	1163	A	G	0.068	1.94E-08
	ASTG7105	31	T	G	0.061	1.46E-09
	ASTG720	430	C	A	0.053	1.27E-06
	ASTG726	99	G	C	0.106	2.62E-07
	ASTG785	321	G	T	0.250	2.06E-07
	ASTG81	362	C	A	0.102	5.30E-08
	ASTG812	1031	A	G	0.061	7.87E-11
	ASTG823	225	A	T	0.070	1.89E-08
	ASTG823	208	A	T	0.069	1.12E-08
	ASTG843	1653	G	A	0.079	2.39E-06
	ASTG8986	503	G	A	0.064	3.48E-10
	ASTG9571	245	T	C	0.054	2.38E-06
	ASTG969	626	T	C	0.082	8.62E-07
	ASTG9701	73	C	T	0.052	2.72E-08
	ASTG9701	54	C	T	0.058	5.56E-09
	ASTG9701	62	G	A	0.058	5.56E-09
	ASTG9899	21	T	C	0.064	2.25E-10
ASTG3011	*ASTG10176*	1594	T	C	0.133	3.93E-07
	ASTG1099	120	C	T	0.076	1.65E-09
	ASTG12259	1730	G	A	0.064	7.88E-07
	ASTG14310	1107	T	G	0.073	3.76E-08
	ASTG14823	3464	G	A	0.071	1.48E-06
	ASTG24897	331	A	G	0.055	1.94E-06
	ASTG25117	18	G	A	0.071	2.09E-07
	ASTG253	223	T	A	0.066	1.70E-09
	ASTG25642	763	T	G	0.098	5.36E-10
	ASTG26379	2054	A	T	0.059	1.99E-06
	ASTG26602	1823	C	A	0.051	5.45E-08
	ASTG26786	333	G	A	0.058	2.50E-06
	ASTG30588	81	G	C	0.075	9.51E-09
	ASTG31348	378	T	C	0.132	9.37E-07
	ASTG31381	2268	A	G	0.141	3.46E-07
	ASTG31921	37	A	G	0.063	1.66E-06
	ASTG3231	13	C	T	0.063	3.36E-07
	ASTG3277	418	T	A	0.105	1.21E-07
	ASTG32822	19	T	A	0.059	1.13E-06
	ASTG32865	1904	C	T	0.071	2.09E-07
	ASTG32865	1865	A	T	0.063	5.84E-08
	ASTG3290	1325	T	G	0.063	2.83E-07

	ASTG33067	2196	A	G	0.112	7.03E-07
	ASTG33495	399	A	G	0.072	3.17E-07
	ASTG33610	1003	A	G	0.115	5.03E-07
	ASTG341	755	C	T	0.098	1.08E-06
	ASTG34206	861	T	A	0.072	2.44E-07
	ASTG34765	1155	T	C	0.123	7.08E-07
	ASTG34765	889	C	A	0.127	2.74E-07
	ASTG34765	523	T	A	0.062	2.74E-07
	ASTG34839	73	G	A	0.076	8.62E-07
	ASTG35120	1184	C	T	0.125	1.58E-06
	ASTG35120	1264	T	C	0.133	7.50E-07
	ASTG35749	41	G	C	0.070	1.38E-06
	ASTG35749	29	A	G	0.068	1.20E-06
	ASTG35856	557	A	G	0.087	2.35E-06
	ASTG36117	270	G	A	0.110	7.25E-07
	ASTG3772	1007	G	A	0.084	1.59E-06
	ASTG3994	344	C	A	0.068	1.14E-08
	ASTG3994	352	C	T	0.068	1.14E-08
	ASTG4626	58	C	T	0.089	4.60E-07
	ASTG4652	708	G	A	0.113	1.45E-06
	ASTG4669	16	A	G	0.062	1.69E-07
	ASTG6213	667	A	G	0.064	2.30E-07
	ASTG6596	1078	T	G	0.053	1.68E-06
	ASTG66	175	A	T	0.099	5.91E-07
	ASTG69	379	A	G	0.190	1.22E-06
	ASTG827	526	A	G	0.052	1.17E-06
	ASTG83	255	T	C	0.063	5.80E-08
	ASTG860	160	T	C	0.051	5.20E-07
	ASTG9	135	A	G	0.145	4.97E-09
	ASTG922	138	T	A	0.067	1.80E-07
	ASTG954	686	C	A	0.108	2.35E-06
	ASTG954	701	G	A	0.108	2.35E-06
	ASTG954	687	A	T	0.100	2.30E-06
ASTG9975	*ASTG1166*	1100	C	T	0.188	6.16E-08
	ASTG1251	645	G	A	0.150	8.92E-07
	ASTG1251	3028	G	A	0.175	8.48E-07
	ASTG1251	285	C	T	0.177	5.89E-08
	ASTG1251	1954	A	G	0.175	6.11E-10
	ASTG136	96	A	C	0.102	6.79E-12
	ASTG1364	667	G	C	0.083	3.87E-07
	ASTG15813	1375	G	A	0.078	1.39E-07
	ASTG1848	90	G	A	0.281	1.15E-07
	ASTG2103	671	C	T	0.081	1.10E-06
	ASTG22942	1395	T	C	0.053	1.69E-06

ASTG25938	50	T	C	0.276	4.12E-07
ASTG25938	51	C	T	0.276	4.12E-07
ASTG2744	942	C	T	0.050	7.14E-08
ASTG28223	555	C	T	0.078	4.23E-08
ASTG3020	1362	G	T	0.154	6.06E-10
ASTG30703	15	G	A	0.077	5.77E-07
ASTG30843	1183	T	C	0.083	2.41E-12
ASTG3099	1100	G	A	0.141	1.99E-06
ASTG3099	1224	A	G	0.233	7.22E-08
ASTG30999	1628	A	G	0.274	1.28E-06
ASTG33394	678	T	C	0.215	8.94E-07
ASTG33394	681	G	C	0.215	8.94E-07
ASTG33394	210	T	G	0.215	8.05E-09
ASTG33508	704	T	C	0.364	1.76E-09
ASTG336	488	G	A	0.283	3.50E-13
ASTG33783	830	G	A	0.242	6.22E-07
ASTG342	427	A	G	0.060	2.09E-07
ASTG34573	709	C	T	0.371	8.08E-07
ASTG34573	1648	A	G	0.364	2.46E-13
ASTG34776	502	G	C	0.067	2.12E-06
ASTG34776	503	A	G	0.067	2.12E-06
ASTG34776	359	G	A	0.066	1.80E-06
ASTG34776	548	A	G	0.295	5.85E-07
ASTG34835	241	A	G	0.282	5.11E-09
ASTG34835	402	G	A	0.282	5.11E-09
ASTG35195	235	T	C	0.203	2.46E-06
ASTG35195	271	A	G	0.154	2.89E-07
ASTG3536	1583	A	T	0.078	4.75E-08
ASTG35417	28	T	C	0.144	2.18E-06
ASTG35417	35	C	A	0.144	2.18E-06
ASTG35427	449	G	A	0.127	1.67E-06
ASTG35476	716	T	C	0.051	5.81E-10
ASTG35679	385	G	A	0.276	7.77E-07
ASTG35924	286	T	C	0.050	1.53E-11
ASTG35924	316	A	G	0.050	1.53E-11
ASTG35924	375	T	C	0.050	1.53E-11
ASTG35955	918	C	T	0.316	1.66E-07
ASTG36032	276	C	G	0.369	2.18E-06
ASTG3737	1386	A	G	0.265	6.34E-09
ASTG459	521	T	G	0.369	1.08E-09
ASTG615	331	G	A	0.435	2.08E-06
ASTG615	415	G	C	0.435	2.08E-06
ASTG7038	957	T	C	0.059	4.87E-07
ASTG810	550	C	G	0.138	7.50E-11

	ASTG830	430	T	G	0.242	1.01E-06
	ASTG830	2495	C	T	0.246	3.69E-07
	ASTG830	2688	C	G	0.246	3.69E-07
	ASTG830	212	A	G	0.215	8.05E-09
	ASTG830	713	C	A	0.215	8.05E-09
	ASTG830	2709	G	T	0.215	8.05E-09
	ASTG830	2736	G	A	0.215	8.05E-09
	ASTG8698	16	C	G	0.053	6.52E-07
	ASTG9009	1952	C	T	0.385	5.14E-08
	ASTG963	469	A	C	0.224	6.73E-07
	ASTG992	1468	A	G	0.117	7.14E-12
	Cluster_132_0	139	T	C	0.259	1.67E-06
	Cluster_132_0	269	A	T	0.204	4.17E-07
	Cluster_132_0	272	A	T	0.204	4.17E-07
ASTG13201	*ASTG151*	259	T	C	0.053	1.26E-06
	ASTG350	31	A	C	0.109	6.37E-07
ASTG28883	*ASTG33067*	1429	A	G	0.070	2.32E-06
	ASTG23014	897	A	G	0.218	3.00E-07
ASTG29757	*ASTG13880*	50	T	C	0.407	1.05E-06
	ASTG1416	209	C	A	0.297	1.14E-06
	ASTG1416	403	T	C	0.109	4.96E-07
	ASTG1416	1779	T	C	0.300	2.03E-08
	ASTG1416	1410	G	A	0.292	1.12E-08
	ASTG1416	184	G	C	0.274	1.70E-13
	ASTG180	782	T	C	0.182	8.42E-08
	ASTG19178	122	G	A	0.081	5.09E-07
	ASTG2103	671	C	T	0.081	2.67E-07
	ASTG25938	50	T	C	0.276	2.48E-06
	ASTG25938	51	C	T	0.276	2.48E-06
	ASTG3020	1362	G	T	0.154	1.86E-06
	ASTG33985	27	C	T	0.240	1.60E-06
	ASTG341	1350	G	A	0.129	1.23E-06
	ASTG3501	1698	A	G	0.204	2.32E-07
	ASTG35195	192	T	G	0.120	2.96E-07
	ASTG35427	321	C	T	0.053	2.51E-07
	ASTG35427	334	T	A	0.053	2.51E-07
	ASTG35427	589	A	G	0.054	2.01E-07
	ASTG35427	266	T	C	0.119	5.76E-08
	ASTG35427	633	T	C	0.125	2.88E-09
	ASTG35427	449	G	A	0.127	5.33E-13
	ASTG35640	730	G	C	0.129	3.80E-07
	ASTG35908	1471	C	T	0.118	2.35E-07
	ASTG433	107	A	G	0.254	1.08E-06
	ASTG780	1257	C	T	0.055	5.70E-08

	Cluster_132_0	483	T	C	0.176	6.60E-09
ASTG29985	NS					
ASTG34481	*Cluster_112_0*	457	A	G	0.220	2.69E-08
	Cluster_112_0	177	C	T	0.094	1.12E-08
	ASTG1047	225	A	G	0.137	2.45E-07
	ASTG1122	237	C	T	0.201	1.57E-06
	ASTG1209	2022	A	G	0.130	1.98E-07
	ASTG1209	2033	A	G	0.132	1.40E-07
	ASTG1296	104	G	A	0.317	1.07E-08
	ASTG1548	734	A	T	0.053	3.13E-09
	ASTG1735	485	G	A	0.092	1.95E-06
	ASTG184	633	C	T	0.103	1.23E-08
	ASTG2240	63	C	T	0.325	1.07E-06
	ASTG233	238	C	A	0.095	6.59E-09
	ASTG23362	266	T	C	0.351	5.70E-07
	ASTG23370	1182	T	C	0.096	2.00E-08
	ASTG25139	147	G	A	0.192	1.92E-07
	ASTG25284	504	C	G	0.364	1.88E-06
	ASTG28138	1251	C	T	0.107	8.87E-09
	ASTG28138	1235	C	T	0.100	4.79E-09
	ASTG28451	59	C	G	0.064	3.54E-07
	ASTG29576	21	A	G	0.204	6.83E-07
	ASTG29576	12	A	G	0.202	4.44E-08
	ASTG29576	24	C	A	0.202	4.44E-08
	ASTG29905	1317	T	C	0.098	3.38E-09
	ASTG30662	1015	C	T	0.061	2.41E-09
	ASTG32612	1620	G	A	0.113	5.12E-07
	ASTG3282	61	G	A	0.064	4.42E-09
	ASTG34573	461	G	A	0.058	2.42E-09
	ASTG34915	51	T	C	0.203	6.42E-08
	ASTG35194	1570	A	G	0.364	1.88E-06
	ASTG35194	735	T	G	0.076	2.51E-07
	ASTG35430	499	T	C	0.097	8.91E-08
	ASTG35731	59	T	G	0.054	6.65E-07
	ASTG35731	411	T	C	0.089	3.02E-07
	ASTG35731	342	C	G	0.051	8.10E-10
	ASTG35867	339	C	T	0.079	1.60E-07
	ASTG35894	348	T	C	0.053	1.65E-06
	ASTG416	581	T	A	0.280	1.41E-06
	ASTG4614	180	T	A	0.080	3.27E-07
	ASTG5153	2989	A	G	0.085	3.93E-07
	ASTG6523	101	T	A	0.063	8.71E-08
	ASTG6523	260	T	C	0.075	1.01E-08
	ASTG6523	763	G	T	0.095	6.59E-09

	ASTG6523	373	A	T	0.071	5.20E-09
	ASTG884	1159	C	T	0.196	2.06E-06
	ASTG9774	61	C	T	0.187	8.36E-07
ASTG34606	*ASTG31313*	1059	C	T	0.058	1.96E-06
	ASTG33120	469	G	C	0.140	9.51E-07
	ASTG34126	123	A	T	0.056	9.09E-08
	ASTG35591	56	C	T	0.136	3.73E-07
	ASTG35862	34	T	C	0.070	7.90E-07
	ASTG543	17	T	C	0.089	1.93E-07
	ASTG922	138	T	A	0.067	1.44E-06
ASTG34835	*ASTG10870*	1278	T	C	0.111	8.80E-07
	ASTG120	495	G	A	0.254	5.50E-07
	ASTG1297	604	T	C	0.241	1.12E-08
	ASTG1297	626	C	T	0.241	1.12E-08
	ASTG1297	636	C	T	0.241	1.12E-08
	ASTG1342	631	A	T	0.054	2.01E-06
	ASTG13880	50	T	C	0.407	2.11E-06
	ASTG19178	122	G	A	0.081	4.56E-12
	ASTG2103	671	C	T	0.081	2.05E-09
	ASTG213	784	T	C	0.095	2.73E-07
	ASTG2403	28	T	C	0.207	8.48E-12
	ASTG25938	50	T	C	0.276	1.91E-06
	ASTG25938	51	C	T	0.276	1.91E-06
	ASTG26662	15	G	A	0.191	2.41E-06
	ASTG30399	300	T	C	0.114	2.73E-07
	ASTG30855	725	T	C	0.164	1.50E-06
	ASTG31287	199	A	C	0.230	8.15E-08
	ASTG32469	1456	T	A	0.142	1.80E-07
	ASTG341	971	T	C	0.136	7.58E-07
	ASTG341	1350	G	A	0.129	3.54E-10
	ASTG3410	522	A	G	0.185	1.60E-06
	ASTG34633	328	C	G	0.333	3.86E-07
	ASTG34633	642	C	T	0.333	3.86E-07
	ASTG34708	588	C	T	0.135	8.01E-07
	ASTG34755	629	T	A	0.057	2.06E-06
	ASTG34759	1476	T	C	0.054	9.53E-09
	ASTG35195	192	T	G	0.120	1.17E-06
	ASTG35283	690	T	C	0.057	2.04E-08
	ASTG355	806	T	C	0.199	1.79E-06
	ASTG35626	347	T	C	0.063	3.46E-10
	ASTG35640	730	G	C	0.129	3.09E-09
	ASTG35908	472	C	T	0.130	2.45E-06
	ASTG35908	473	C	A	0.130	2.45E-06
	ASTG35908	1068	T	C	0.147	5.89E-08

	ASTG3865	625	T	G	0.207	8.48E-12
	ASTG4201	1898	A	T	0.095	6.48E-07
	ASTG5360	211	C	T	0.094	2.32E-08
	ASTG5371	16	G	T	0.176	2.22E-06
	ASTG5784	452	C	T	0.254	9.12E-08
	ASTG689	528	G	A	0.283	7.74E-09
ASTG35290	*ASTG1003*	554	C	A	0.313	2.00E-09
	ASTG1044	194	A	G	0.100	2.10E-06
	ASTG1044	160	G	C	0.084	3.79E-08
	ASTG10870	2697	G	A	0.109	2.03E-07
	ASTG1091	284	A	C	0.264	3.23E-08
	ASTG1091	343	A	G	0.264	3.23E-08
	ASTG1091	384	G	A	0.264	3.23E-08
	ASTG11167	18	C	T	0.098	4.93E-08
	ASTG1209	2346	G	A	0.104	1.16E-06
	ASTG1215	1632	T	C	0.219	6.74E-08
	ASTG12308	512	C	T	0.059	5.49E-07
	ASTG13045	1410	T	G	0.075	1.07E-10
	ASTG13226	27	C	T	0.099	7.02E-07
	ASTG13483	29	T	G	0.130	2.21E-07
	ASTG15354	1043	T	A	0.222	6.89E-07
	ASTG1653	342	G	C	0.099	1.71E-10
	ASTG17758	78	T	A	0.067	1.01E-06
	ASTG19266	35	A	G	0.095	4.41E-08
	ASTG20087	12	A	G	0.067	4.53E-11
	ASTG206	214	A	G	0.057	5.38E-07
	ASTG216	158	G	A	0.062	2.12E-07
	ASTG2226	1861	A	G	0.169	4.57E-08
	ASTG2226	1862	T	C	0.169	4.57E-08
	ASTG2226	1839	C	T	0.169	2.86E-08
	ASTG2282	887	G	T	0.080	3.17E-07
	ASTG2282	920	G	T	0.080	3.17E-07
	ASTG23918	14	C	T	0.072	1.29E-06
	ASTG241	399	A	G	0.088	4.78E-07
	ASTG24863	1131	A	G	0.062	8.37E-07
	ASTG2677	1509	C	T	0.119	1.82E-07
	ASTG2679	1748	C	T	0.142	1.37E-07
	ASTG2812	828	G	T	0.139	1.79E-07
	ASTG29101	152	T	G	0.222	6.89E-07
	ASTG29101	166	A	G	0.222	6.89E-07
	ASTG30900	1085	C	G	0.222	6.89E-07
	ASTG30992	920	G	T	0.218	1.40E-06
	ASTG31231	76	C	A	0.062	2.04E-07
	ASTG31288	434	C	T	0.165	3.35E-07

ASTG31288	443	T	G	0.165	3.35E-07
ASTG31395	387	A	G	0.052	5.68E-07
ASTG31420	35	C	T	0.111	6.89E-07
ASTG31420	48	G	T	0.111	6.89E-07
ASTG32018	606	A	C	0.112	2.09E-06
ASTG32018	322	T	C	0.106	1.61E-06
ASTG32076	336	G	A	0.066	7.14E-08
ASTG32426	918	A	G	0.118	3.78E-07
ASTG32623	283	G	A	0.222	6.89E-07
ASTG32623	426	C	T	0.222	6.89E-07
ASTG32704	562	G	A	0.130	1.17E-06
ASTG33120	327	T	C	0.127	9.24E-07
ASTG334	96	C	A	0.061	1.83E-10
ASTG33809	584	T	C	0.054	6.14E-10
ASTG34208	667	G	A	0.083	6.46E-12
ASTG34311	1158	A	G	0.068	2.78E-08
ASTG34411	806	A	G	0.072	6.18E-08
ASTG34440	647	T	A	0.214	1.09E-10
ASTG34440	662	A	G	0.214	1.09E-10
ASTG34573	737	C	T	0.262	1.11E-06
ASTG34741	66	G	A	0.091	2.90E-08
ASTG34758	114	C	T	0.136	3.56E-07
ASTG34915	590	T	A	0.131	4.31E-13
ASTG34937	283	A	T	0.115	4.83E-07
ASTG34956	648	T	A	0.087	2.85E-08
ASTG35184	224	A	G	0.087	2.34E-06
ASTG35194	704	G	A	0.057	5.65E-09
ASTG35290	57	A	G	0.152	8.62E-07
ASTG35323	272	A	G	0.108	2.13E-06
ASTG35378	490	T	C	0.222	6.89E-07
ASTG35476	716	T	C	0.051	1.76E-06
ASTG35570	532	C	A	0.163	5.31E-09
ASTG3564	583	G	A	0.107	3.58E-07
ASTG35750	146	G	T	0.065	3.92E-07
ASTG35750	404	C	T	0.141	1.66E-07
ASTG35756	35	G	T	0.073	1.09E-07
ASTG35837	20	G	A	0.083	8.68E-07
ASTG35848	624	T	C	0.155	1.99E-06
ASTG35854	692	C	T	0.238	6.03E-07
ASTG35925	474	C	T	0.175	5.91E-08
ASTG35925	476	A	G	0.167	5.47E-08
ASTG35995	395	T	C	0.069	4.61E-08
ASTG36001	587	C	T	0.187	7.83E-07
ASTG36001	563	G	A	0.181	1.41E-07

	ASTG36001	161	A	G	0.180	8.04E-08
	ASTG36001	133	C	G	0.149	7.16E-08
	ASTG36012	764	G	A	0.067	5.14E-07
	ASTG36096	432	T	C	0.321	2.27E-08
	ASTG36096	478	T	A	0.321	2.27E-08
	ASTG3890	189	T	C	0.092	1.09E-06
	ASTG440	16	C	T	0.175	7.55E-07
	ASTG4880	43	C	A	0.104	1.15E-08
	ASTG4913	147	A	T	0.112	2.38E-08
	ASTG4976	1125	T	G	0.100	1.89E-06
	ASTG5073	41	A	G	0.333	1.89E-09
	ASTG5397	1269	A	T	0.103	3.91E-10
	ASTG6488	1111	A	T	0.104	1.34E-06
	ASTG6809	170	A	G	0.133	1.74E-06
	ASTG7389	21	A	G	0.175	1.54E-10
	ASTG741	106	G	A	0.086	2.01E-06
	ASTG810	550	C	G	0.138	2.50E-06
	ASTG8644	226	G	A	0.298	1.92E-10
	ASTG882	303	C	T	0.185	2.75E-07
	ASTG920	824	G	A	0.279	5.76E-09
	ASTG93	282	A	T	0.105	1.49E-06
	ASTG93	301	G	C	0.087	4.02E-07
	ASTG93	453	T	C	0.086	2.97E-08
	ASTG969	2097	T	C	0.241	3.52E-09
	ASTG969	2110	T	G	0.232	1.76E-09
	ASTG969	2096	C	T	0.233	4.92E-11
ASTG36040	*ASTG2012*	1087	A	T	0.403	1.42E-08
	ASTG2115	617	C	T	0.115	5.03E-13
	ASTG32367	731	G	A	0.118	1.71E-10
	ASTG32625	275	A	G	0.259	4.59E-08
	ASTG32625	328	A	C	0.259	4.59E-08
	ASTG34510	751	C	T	0.190	4.01E-08
	ASTG34510	68	C	T	0.246	3.58E-08
	ASTG492	37	C	T	0.303	6.30E-08
ASTG36172	NS					

Table S6 Suggestive loci associated with three garlic bulb-yield traits ($P < 5\times10^{-5}$)

Traits	Transcript	SNP position	REF	ALT	maf	P value
BD	ASTG1209	2033	A	G	0.132	4.94E-06
	ASTG155	208	A	G	0.079	1.81E-07
	ASTG2361	802	A	G	0.093	3.85E-05
	ASTG32176	34	C	T	0.075	2.56E-05
	ASTG32604	327	C	T	0.152	1.87E-05
	ASTG33002	151	A	G	0.178	9.92E-06
	ASTG33285	708	C	T	0.086	1.44E-06
	ASTG33350	26	A	G	0.240	9.83E-06
	ASTG34606	955	G	A	0.419	5.61E-05
	ASTG35177	460	C	T	0.051	3.94E-05
	ASTG4710	119	G	T	0.069	4.94E-06
	ASTG780	1373	G	A	0.060	1.96E-05
BW	ASTG10837	823	A	G	0.069	1.27E-05
	ASTG10870	1690	A	G	0.191	1.86E-06
	ASTG1095	1587	C	T	0.250	3.57E-06
	ASTG120	495	G	A	0.254	3.57E-06
	ASTG1209	2033	A	G	0.132	8.88E-07
	ASTG13880	50	T	C	0.407	3.57E-06
	ASTG15393	12	A	G	0.077	1.63E-05
	ASTG155	208	A	G	0.079	5.30E-09
	ASTG1604	397	A	G	0.069	1.27E-05
	ASTG16546	881	A	T	0.077	9.42E-07
	ASTG1690	306	A	T	0.136	5.09E-06
	ASTG18710	262	C	T	0.078	1.90E-05
	ASTG1912	528	C	T	0.246	4.44E-07
	ASTG19191	2812	T	C	0.167	7.95E-06
	ASTG19471	36	C	G	0.072	2.27E-05
	ASTG2103	671	C	T	0.081	2.56E-06
	ASTG21946	29	A	T	0.168	1.12E-05
	ASTG22087	3191	A	T	0.265	2.77E-05
	ASTG22711	84	A	G	0.167	3.08E-07
	ASTG23362	292	G	A	0.360	3.57E-06
	ASTG241	929	G	C	0.151	2.30E-05
	ASTG25369	848	A	C	0.268	2.70E-05
	ASTG28223	555	C	T	0.078	2.69E-05
	ASTG28946	19	C	T	0.141	1.27E-05
	ASTG29913	1491	G	A	0.219	5.73E-07
	ASTG30540	15	A	C	0.081	2.56E-06
	ASTG31122	268	C	G	0.167	4.74E-05
	ASTG32176	34	C	T	0.075	2.70E-05
	ASTG32604	327	C	T	0.152	5.12E-06
	ASTG33002	151	A	G	0.178	4.79E-08

	ASTG33285	708	C	T	0.086	5.08E-05
	ASTG33328	746	C	T	0.311	2.93E-06
	ASTG33350	26	A	G	0.240	1.15E-05
	ASTG33394	210	T	G	0.215	5.73E-07
	ASTG33450	546	A	T	0.226	5.73E-07
	ASTG33562	553	A	G	0.245	3.57E-06
	ASTG33586	1816	A	G	0.149	2.04E-05
	ASTG33783	830	G	A	0.242	2.70E-05
	ASTG34094	741	A	G	0.377	3.57E-06
	ASTG341	581	G	T	0.117	2.70E-06
	ASTG34209	695	G	A	0.230	3.57E-06
	ASTG34285	1569	A	T	0.375	7.00E-07
	ASTG34835	402	G	A	0.282	1.94E-06
	ASTG3536	1583	A	T	0.078	1.28E-05
	ASTG35405	276	T	C	0.071	1.27E-05
	ASTG35417	35	C	A	0.144	1.90E-06
	ASTG35474	394	A	T	0.141	2.08E-05
	ASTG35848	558	A	G	0.258	2.70E-06
	ASTG35854	99	T	A	0.233	5.73E-07
	ASTG36032	276	C	G	0.369	4.21E-05
	ASTG36096	511	G	A	0.245	1.39E-05
	ASTG361	537	A	G	0.073	4.93E-05
	ASTG3853	19	C	T	0.084	1.01E-05
	ASTG433	107	A	G	0.254	1.61E-05
	ASTG4595	602	C	A	0.173	3.57E-06
	ASTG4710	119	G	T	0.069	8.88E-07
	ASTG6147	232	G	A	0.355	3.81E-05
	ASTG8140	1827	T	C	0.096	3.28E-05
	ASTG830	2736	G	A	0.215	5.73E-07
	Cluster_132_0	483	T	C	0.176	5.36E-06
CN	ASTG10009	466	T	C	0.063	3.67E-05
	ASTG10870	3161	G	A	0.059	8.24E-06
	ASTG1099	213	A	G	0.073	2.21E-06
	ASTG1118	31	A	G	0.078	1.30E-06
	ASTG12548	1482	A	T	0.058	5.59E-06
	ASTG12568	779	G	T	0.109	1.97E-05
	ASTG13201	18	T	A	0.086	2.01E-05
	ASTG1416	1408	T	C	0.153	5.85E-06
	ASTG1470	1055	T	C	0.090	2.74E-05
	ASTG14831	17	A	C	0.050	3.91E-05
	ASTG1525	415	C	T	0.062	4.25E-08
	ASTG178	81	G	A	0.051	1.28E-05
	ASTG19715	3179	G	A	0.104	4.14E-05
	ASTG207	138	G	T	0.063	6.42E-10

ASTG208	183	G	A	0.063	1.03E-06
ASTG2142	253	C	T	0.065	5.03E-06
ASTG22147	52	C	T	0.054	3.08E-06
ASTG23088	43	C	T	0.064	1.03E-05
ASTG2361	703	A	G	0.115	2.55E-05
ASTG2382	168	T	A	0.068	1.60E-06
ASTG25083	2863	T	C	0.066	1.09E-06
ASTG26122	139	G	A	0.077	3.27E-05
ASTG26171	141	G	C	0.082	3.92E-05
ASTG26534	1864	C	T	0.075	4.60E-06
ASTG28317	408	C	T	0.123	1.83E-05
ASTG2881	1678	A	G	0.065	1.47E-05
ASTG28883	572	T	C	0.073	1.03E-07
ASTG29757	52	T	G	0.051	1.11E-06
ASTG29860	203	C	T	0.098	1.94E-05
ASTG29985	682	C	T	0.130	1.87E-06
ASTG3011	152	T	A	0.098	4.87E-08
ASTG30497	42	G	T	0.066	2.44E-05
ASTG30971	13	A	G	0.069	5.16E-06
ASTG30983	1923	T	C	0.078	1.15E-05
ASTG311	778	T	A	0.129	1.32E-05
ASTG31175	450	T	C	0.143	3.16E-05
ASTG31304	519	T	C	0.068	4.81E-06
ASTG32018	478	G	A	0.116	2.60E-06
ASTG32580	1917	G	A	0.149	2.87E-05
ASTG32586	1797	T	A	0.068	1.25E-05
ASTG32612	2213	G	A	0.145	1.25E-05
ASTG32928	1387	T	C	0.074	4.36E-05
ASTG33222	572	T	C	0.107	1.73E-05
ASTG33250	363	T	C	0.082	7.40E-06
ASTG33838	1549	A	G	0.118	1.55E-05
ASTG3387	766	C	T	0.142	1.12E-05
ASTG34311	1158	A	G	0.068	4.65E-06
ASTG3440	721	G	A	0.107	2.52E-05
ASTG34481	1035	T	A	0.188	2.94E-09
ASTG34563	1491	A	T	0.103	3.29E-05
ASTG34606	1228	T	C	0.089	1.77E-06
ASTG34716	505	T	A	0.109	1.25E-05
ASTG34765	174	A	G	0.107	6.25E-06
ASTG34837	708	A	T	0.063	8.19E-06
ASTG3485	121	G	A	0.073	3.54E-05
ASTG34894	475	T	C	0.125	6.84E-06
ASTG34956	398	A	G	0.081	1.31E-05
ASTG35290	292	C	A	0.083	3.63E-07

ASTG35311	488	C	T	0.083	4.25E-05
ASTG35328	25	G	A	0.058	1.95E-09
ASTG35329	736	C	T	0.140	2.78E-05
ASTG35406	28	T	G	0.115	3.56E-08
ASTG35422	337	T	C	0.088	9.07E-08
ASTG35437	53	T	A	0.188	1.80E-05
ASTG35440	504	A	G	0.108	2.82E-05
ASTG35593	1823	T	C	0.070	4.26E-05
ASTG35650	949	T	C	0.157	4.96E-05
ASTG35686	149	A	G	0.081	4.45E-06
ASTG35767	62	C	T	0.096	1.92E-05
ASTG35804	25	T	A	0.092	4.06E-05
ASTG35863	884	G	T	0.086	4.68E-05
ASTG35918	54	A	G	0.063	7.20E-06
ASTG35935	109	A	C	0.153	1.19E-06
ASTG35951	293	G	A	0.118	9.54E-10
ASTG36029	752	C	G	0.082	3.32E-05
ASTG36032	99	G	T	0.100	1.88E-05
ASTG36040	445	A	G	0.137	3.17E-08
ASTG36061	503	C	G	0.090	4.76E-06
ASTG36172	476	G	A	0.069	2.28E-06
ASTG4210	215	G	A	0.070	8.90E-06
ASTG4387	385	C	T	0.203	1.23E-05
ASTG4427	160	T	C	0.170	1.69E-06
ASTG4820	207	A	G	0.068	3.15E-05
ASTG49	42	T	C	0.151	2.99E-05
ASTG50	197	C	T	0.065	1.69E-05
ASTG5030	88	C	G	0.059	1.74E-05
ASTG5261	488	A	T	0.052	9.40E-06
ASTG628	1154	C	T	0.069	2.06E-05
ASTG639	106	C	T	0.090	4.71E-09
ASTG652	2079	G	A	0.073	4.69E-05
ASTG661	802	G	A	0.063	7.97E-06
ASTG6809	668	C	T	0.072	2.72E-05
ASTG7586	785	T	A	0.091	1.92E-06
ASTG801	263	C	T	0.111	2.23E-05
ASTG823	239	T	C	0.065	4.82E-06
ASTG8311	1806	G	A	0.065	4.37E-05
ASTG832	1181	C	T	0.130	4.61E-05
ASTG8438	51	C	T	0.098	4.64E-05
ASTG9152	42	A	T	0.058	4.06E-06
ASTG977	272	T	C	0.057	8.66E-09
ASTG9975	1303	A	C	0.058	4.50E-09
